# Human Enterovirus Genotype C104, China

**DOI:** 10.3201/eid1904.121435

**Published:** 2013-04

**Authors:** Zichun Xiang, Zhengde Xie, Zhong Wang, Lili Ren, Yan Xiao, Linlin Li, Guy Vernet, Gláucia Paranhos-Baccalà, Kunling Shen, Jianwei Wang

**Affiliations:** Ministry of Health Key Laboratory of Systems Biology of Pathogens, Beijing, People’s Republic of China (Z. Xiang, L. Ren, L. Li, J. Wang);; Institute of Pathogen Biology, Beijing (Z. Xiang, L. Ren, Y. Xiao, L. Li, J. Wang);; Beijing Children’s Hospital, Beijing (Z. Xie, K. Shen); Peking Union Medical College Hospital, Beijing (Z. Wang);; Fondation Mérieux, Lyon, France (G. Vernet, G. Paranhos-Baccalà)

**Keywords:** human enterovirus, viruses, EV-C104, respiratory tract infections, China

**To the Editor:** Human enteroviruses (EVs) are small, nonenveloped RNA viruses belonging to the family *Picornaviridae*. Approximately 100 EV genotypes have been identified. Recently, EV68 epidemics in respiratory tract infections (RTIs) have been reported worldwide (*1**,**2*). Moreover, rarely detected EVs (e.g., EV-C104 and EV-C109) have been increasingly identified in patients with RTIs (*3**–**7*), indicating a possible association of EVs with respiratory syndromes.

Little is known about the role of EV-C104 in RTIs. EV-C104 has been reported in 3 countries: Switzerland (7 children with pneumonia or otitis media) (*3*), Italy (3 adults and 4 children with RTIs) (*4**,**7**),* and Japan (1 adult with an upper RTI [URTI](*5*). We report additional EV-C104 strains in 4 children with lower RTIs and in 1 adult with a URTI in China.

To identify EV infections, we collected nasopharyngeal aspirates from 3,108 children (1,963 boys) ≤14 years of age (median age 12 months; age range 0.3–168 months) who had lower RTIs at admission to Beijing Children’s Hospital during March 2007–February 2012. Throat and nasal swab specimens were also collected from 9,232 adults (4,140 men) ≥ 15 years of age (median age 35.3 years; age range 15–97 years) with acute RTIs who received treatment at Peking Union Medical College Hospital during August 2006–February 2012. All samples were screened for influenza virus, parainfluenza virus type 1–4, respiratory syncytial virus (RSV), coronaviruses (229E, NL63, HKU1, and OC43), metapneumovirus, adenovirus, rhinovirus, bocavirus, and EVs (*8*). Overall, 37 (1.2%) children and 158 (1.7%) adults were positive for EV.

Because we could not amplify EV-C104 by using primers specific for the viral protein (VP) 1 region (*9*), we used a reverse transcription PCR to amplify the 5′-untranslated region/VP4/VP2 gene (*10*). Amplicons of ≈600 bp were obtained for samples from 5 patients: 4 boys 2–11 months of age (BCH2859A, BCH2892A, BCH2894A, and BCH3034A) and a 30-year-old man (PUMCH12286). BLAST analysis (www.ncbi.nlm.nih.gov) of sequences of these amplicons showed that the 590-nt sequences had 94.0% identity with that of the EV-C104 prototype strain CL-12310945 (EU840733). The amplicon contained 155 nt in the 5′-untranslated region, 207 nt in VP4, and 228 nt in VP2. Phylogenetic analysis of VP4/VP2 sequences showed that the 5 sequences obtained in this study (GenBank accession nos. JX560522–JX560526) belonged to genotype EV-C104 within the EV-C species (Figure).

**Figure:**
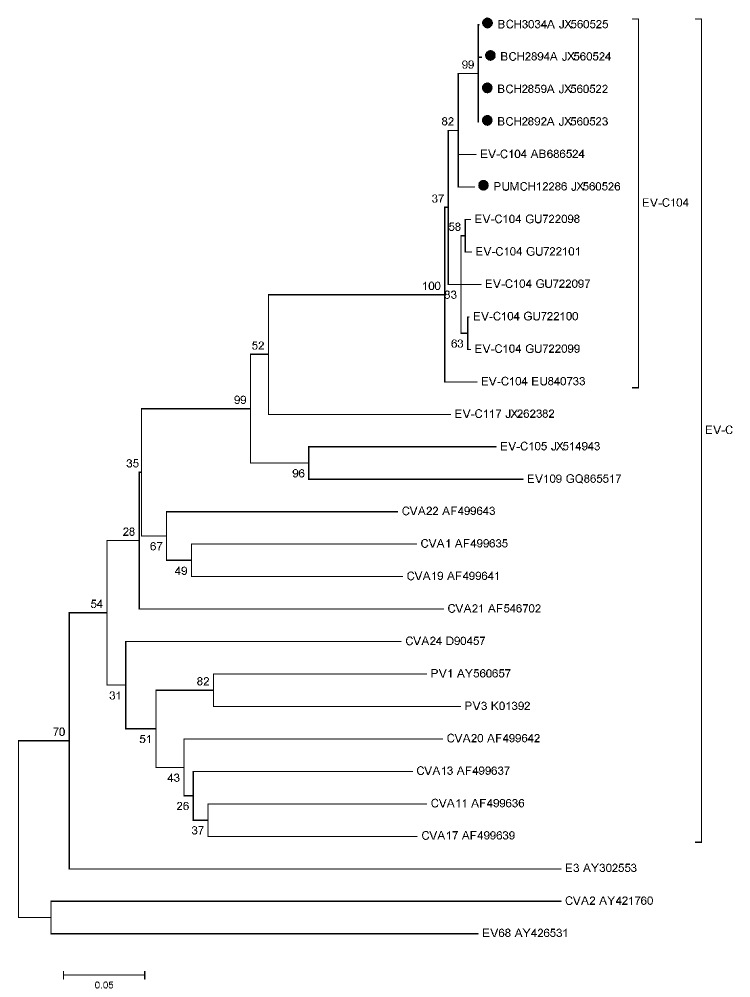
Phylogenetic tree of human enteroviruses (EVs) for nucleotide sequences of the viral protein (VP) 4/VP2 gene region (435 nt, corresponding to nt positions 654–1,088 of EV-C104 prototype strain CL-12310945 [EU840733]), People’s Republic of China, March 2007–February 2012. The tree was generated with 1,000 bootstrap replicates. Neighbor-joining analysis of targeted nucleotide sequence was performed by using the Kimura 2-parameter model with the Molecular Evolutionary Genetics Analysis software version 4.0 (www.megasoftware.net/). Each strain detected in this study is indicated by a black circle and a specific identification code (BCH/PUMCH), followed by the patient number. Enterovirus 68, cocksackievirus (CV) A2, and echovirus (E) 3 (GenBank accession nos. AY426531, AY421760, and AY302553) were used as outgroups. PV, poliovirus. EV-C denotes the EV species to which EV-C104 belongs. Scale bar indicates evolutionary distance.

Virus isolation for EV-C104 with Vero and H1-HeLa cells was unsuccessful. Although we screened 591, 797, 459, 664 and 597 samples from children for 5 consecutive years and 1,765, 1,978, 1,350, 1,562, 1,573, and 1,004 samples from adults for 6 consecutive years, we did not detect EV-C104 strains until November 2011–February 2012.

Nucleotide identity of the EV-C104 sequences from this study was 99.5%–100% among BCH strains and 97.7%–98.0% between the PUMCH strain and the BCH strains. Deduced amino acid sequences in VP4/VP2 among the BCH strains were identical, albeit for 1 aa difference for the PUMCH strain (BCH strains had Pro^110^, but the PUMCH strain had Leu^110^, which was consistent with strains detected in children and adults in Italy). Deduced amino acid sequences for all 5 strains isolated in this study had 97.9%–100.0% identity with those from Switzerland, Italy, and Japan. BCH strains were community acquired because these 4 patients came from different cities and were admitted to different wards on different dates.

The 4 EV-C104–positive boys all had fever and cough for >10 days before their hospitalization. Chest radiographs showed increased lung markings or patchy shadows diagnosed as pneumonia or bronchopneumonia. RSV or adenovirus was also detected in 3 of the boys. The fourth boy was positive for parainfluenza virus type 1, adenovirus, and bocavirus. Clinical outcomes for all 4 children were favorable. The EV-C104–positive man had fever, chills, pantalgia, and expectoration for 1 day before a URTI was diagnosed. EV-C104 was the only virus detected in this patient.

We compared relative viral loads for all viruses in the 5 patients and quantified viral load of EV-C104 and other viruses by using real-time PCR (methods available upon request). Median viral load in the 5 patients was 2.4 × 10^6^ RNA copies/mL (range 5.6 × 10^4^–7.0 × 10^6^ copies RNA/mL (Table, Appendix).

**Table:** Viral load of EV C104 and other viruses in 5 patient samples, People’s Republic of China, March 2007–February 2012*

Patient sample	Virus and viral load (RNA or DNA copies/mL)				
	EV-C104	RSV	Adv	PIV1	HBoV
BCH2859A	7.0 × 10^6^	ND	4.5 × 10^10^	1.2 × 10^7^	3.9 × 10^3^
BCH2892A	5.6 × 10^4^	1.0 × 10^6^	1.1 × 10^9^	ND	ND
BCH2894A	1.7 × 10^5^	ND	7.8 × 10^9^	ND	ND
BCH3034A	2.4 × 10^6^	3.3 × 10^8^	ND	ND	ND
PUMCH12286	3.9 × 10^6^	ND	ND	ND	ND

*EV-C104, enterovirus C104; RSV, respiratory syncytial virus; AdV, human adenovirus; PIV1, human parainfluenza virus type 1; HBoV, human bocavirus; ND, not detected.

Overall, we found few (5/12,340) EV-C104–positive specimens. All EV-C104–positive children were co-infected with RSV or adenoviruses (high viral loads) in our study. The role of EV-C104 in RTIs needs to be further studied. Nevertheless, the finding of EV-C104–positive adults with high viral loads in China (3.9 × 10^6^ RNA copies/mL) and Italy (2.0 × 10^6^ RNA copies/mL) (*7*) indicates a possible association between EV-C104 with RTIs. Our data also confirm a wide distribution of EV-C104.
